# Evaluation of routine coagulation profiles and hematological markers in patients with extremity sarcomas: A cohort study

**DOI:** 10.1016/j.htct.2026.106542

**Published:** 2026-09-20

**Authors:** Giovanni Francisco Lovato, João Pedro Rigoletto Penteado, Mayara Branco e Silva, Silmara Aparecida Lima Montalvão, Stephany Cares Hubers, Joyce Maria Annichino Bizzacchi, Guilherme Grissi Mouraria, Maurício Etchebehere

**Affiliations:** a Faculdade de Ciências Médicas, UNICAMP, Campinas, SP, Brazil; b Serviço de Ortopedia e Traumatologia, Complexo Hospitalar Prefeito Edivaldo Orsi (Hospital Ouro Verde), Campinas, SP, Brazil; c Departamento de Ortopedia, Reumatologia e Traumatologia, Faculdade de Ciências Médicas, UNICAMP, Campinas, SP, Brazil; d Hemocentro, UNICAMP, Campinas, SP, Brazil; e Departamento de Clínica Médica, Faculdade de Cências Médicas, UNICAMP, Campinas, SP, Brazil

Dear editor,

Cancer is a leading cause of global mortality, often associated with systemic hemostatic alterations including thrombosis and hypercoagulability [1]. Tumor cells interact with coagulation factors and platelets, expressing tissue factor (TF) to modify the microenvironment, thereby promoting progression and metastasis [2]. Beyond thromboembolic risks, parameters such as prothrombin time (PT), activated partial thromboplastin time (aPTT), and fibrinogen are emerging as potential indicators of tumor aggressiveness and prognosis [3,4]. Furthermore, blood count indices reflecting thromboinflammation, such as platelet count and platelet-to-neutrophil ratio (PNR), have been implicated in the tumor microenvironment dynamics of various solid tumors.

In sarcomas, these mechanisms remain poorly defined. While shortened PT and aPTT often signal hypercoagulability in other malignancies, their specific prognostic value in bone and soft tissue sarcomas requires further investigation [5,6]. We analyzed baseline PT, aPTT, fibrinogen, and complete blood count (CBC) parameters in sarcoma patients to evaluate their association with clinical outcomes and long-term prognosis [7].

This prospective cohort (2019–2021) at Hospital de Clínicas-Unicamp included 14 patients (age: ≥18) with bone or soft tissue sarcomas of the extremities. Peripheral blood samples were obtained via standard venipuncture at the time of admission, strictly prior to the initiation of any therapeutic intervention (including surgical resection, chemotherapy, or radiotherapy) to ensure baseline comparability. Inclusion required histopathological confirmation, a complete baseline coagulogram (PT, aPTT) prior to treatment, and at least 12 months follow-up. Visceral, retroperitoneal, and craniofacial cases were excluded.

Outcomes included Stage IV disease at admission, subsequent metastasis, recurrence, and mortality. Statistical analysis utilized Mann-Whitney U and Shapiro-Wilk tests for continuous variables, while Fisher’s exact test assessed categorical associations (p < 0.05). Odds ratios (OR) and 95% confidence intervals (95% CI) were calculated where applicable. The study was approved by the local Ethics Committee (CAAE: 87,212,318.7.0000.5404).

The study included 14 patients (mean age: 53.21 years; 92.9% female) with soft tissue (64.3%) or bone (35.7%) sarcomas, primarily of the lower limbs. Bone sarcomas were significantly associated with higher mortality (80%vs. 11.1%; p = 0.023). Over a 3-year follow-up, two patients (14.3%) had local recurrence and five (35.7%) died (overall survival: 64.3%). Age did not correlate with adverse events (p = 0.82).

PT was altered in only one patient (7.1%), who survived without adverse events (p = 1.0). For aPTT, three patients (21.4%) showed abnormalities (two with adverse events; OR: 1.12; 95% CI: 0.08–16.3; p = 1.0).

Comparative analysis between patients with and without metastatic disease showed no significant differences in hematological or coagulogram profiles (Table 1). The comparison between groups with and without adverse events confirmed no significant differences regarding age or any specific coagulation parameters (Table 2).

**Table 1:** Comparison of hematological parameters, coagulation and clinical outcomes between patients with and without metastasis.

Variable	Without metastasis (n = 9)	With metastasis (n = 5)	p-value
Platelet count (x 10³/µL) – median ± SD	307.78 ± 67.10	298.60 ± 107.96	0.85
PT (seconds) – median ± SD	12.03 ± 1.14	12.44 ± 0.98	0.52
aPTT (seconds) – median ± SD	27.81 ± 3.81	26.24 ± 3.04	0.48
Fibrinogen (mg/dL) – median ± SD	462.00 ± 206.58 (n = 7) *	560.00 ± 388.42 (n = 3) *	1.00
D-dimer (ng/mL) – median ± SD	797.50 ± 826.53 (n = 8) *	679.00 ± 583.38 (n = 4) *	0.933
Death - n (%)	1 (11.1)	4 (80.0)	0.023
Recurrence - n (%)	1 (11.1)	1 (11.1)	1.00

SD: Standard deviation.

*Fibrinogen (n = 10) and d-dimer (n = 12) data were available for fewer patients than the other hematological parameters (n = 14) because blood samples were not collected for these assays in all patients.

**Table 2:** P-values obtained in the comparison of variables with the occurrence of adverse events.

Variable	Adverse event	n	Mean	Standard Deviation	p-value
Age (years)	No Yes	8 6	53.88 52.33	15.61 16.11	0.860
PT (seconds)	No Yes	8 6	12.05 12.35	1.22 0.90	0.623
Fibrinogen (mg/dL) *	No Yes	7 3	462.00 560.00	206.58 388.42	1.000
Platelet count (x10³/µL)	No Yes	8 6	303.88 305.33	70.63 97.96	0.950
aPTT (seconds)	No Yes	8 6	27.10 27.40	3.45 3.93	0.897
D-dimer (ng/mL) *	No Yes	8 4	797.50 679.00	826.53 583.38	0.933
Any hematological alteration	Any adverse event	—	—	—	1.000

*Fibrinogen (n = 10) and d-dimer (n = 12) data were available for fewer patients than the other hematological parameters (n = 14) because blood samples were not collected for these assays in all patients.

Our findings indicate that baseline coagulogram parameters were not significantly associated with recurrence, metastasis, or mortality in sarcoma patients. These results partially contrast with reports in the literature suggesting that coagulation activation in the tumor microenvironment drives progression in various solid tumors [1].

In this cohort, only clinical variables reached significance. Bone sarcoma (p = 0.023) and metastatic disease at admission (p = 0.023) were significantly associated with mortality. This reinforces the aggressive nature of bone sarcomas and the role of metastasis as a primary negative prognostic marker, even in small samples.

Baseline fibrinogen and d-dimer levels were also evaluated for their potential association with metastatic disease and adverse clinical outcomes. Although patients with metastasis and those who experienced adverse events tended to present higher fibrinogen levels, these differences were not significant. Similarly, d-dimer levels did not differ significantly according to metastatic status or the occurrence of adverse events.

The lack of correlation may stem from the low sensitivity of conventional PT and aPTT in detecting discrete hemostatic changes. More specific markers, such as tissue factor or thrombin generation assays, might offer higher sensitivity [6]. Furthermore, a single baseline measurement may miss dynamic changes occurring during clinical progression. More specific viscoelastic or molecular markers, such as tissue factor and thrombin generation, might offer higher prognostic sensitivity.

Our primary limitation is the small cohort (n = 14), which restricts statistical power and may obscure histological heterogeneity. Furthermore, data collection was constrained to 2019–2021 due to COVID-19 pandemic institutional adjustments and academic timeframe limitations, preventing the inclusion of patients from 2022 to 2025.

Regarding the valuable suggestion to include fibrinogen and the PNR, we were able to incorporate fibrinogen and d-dimer into the present analysis. However, the PNR could not be evaluated because differential leukocyte counts, including neutrophil counts, were not collected as part of the original study protocol. Consequently, the absence of PNR data remains a limitation that should be addressed in future prospective studies.

Asanuma et al. observed that fibrinolysis markers might reflect disease status more accurately than basic coagulation parameters in soft tissue sarcomas. Notably, these authors reported the same limitation: the need for larger cohorts to validate preliminary findings [8].

In conclusion, while clinical staging and tumor type remain fundamental for risk stratification, baseline routine coagulogram values alone do not appear to be reliable independent predictors of prognosis in sarcoma patients.

## Conflicts of interest

No conflicts of interest.
